# Nature’s Demands

**Published:** 1890-06

**Authors:** 


### HALL'S
### JOURNAL OF HEALTH

TRUTH DEMANDS NO SACRIFICE; ERROR CAN MAKE NONE.

Vol. 37. JUNE, 1890. No. 6.

### NATURE'S DEMANDS.

All parts of the human organism have their allotted functions, which require to be continually exercised in order to maintain their efficiency. Nature is a jealous mother, but those who heed her admonitions, have little cause of complaint. In her vast economy, nothing is made in vain ; nothing but has its uses. Sleeping or waking, the heart must never pause even for a moment in its task of forcing the warm blood currents through their channels in an unceasing round ; nor will the lungs be permitted the least respite in their work of vivification through the respiratory functions.

With every physical or mental exertion there is an expenditure of structure—a waste which must be replenished. The innumerable cells of which the body is composed are constantly wearing away, and new ones are as constantly forming to supply the deficiency. Like the cells of an electric battery, which they resemble functionally, they require to be continually re-established, and it is only when the body loses the capacity for this, that death naturally ensues. There is a close relationship between all the organs of the body, mental as well as physical. The mind cannot be strong and healthy in a weak and unhealthy body, for the one is sure to sympathize with the other, hence the importance of preserving a proper equilibrium of all the parts. This means that no one part should be cultivated or trained at the expense of another. There are very many exercises and diversions not only useful in themselves, but highly important, which, if indulged in to excess, are sure to work injury.

There are also trades and professions, a devotion to which, quicken some of the organs at the expense of others. This, although contrary to the highest development, is scarcely to be avoided under our present social system, where the callings of a lifetime take so many directions independently of others. But with all this, there is little or no excuse for an individual of ordinary capacity to neglect his higher faculties, whatever may be the nature of his daily employments. Any organ or faculty will resent this neglect by slowly dying out beyond recovery. The Hindoo zealot, whose misconceived piety induces him to point a hand continually heavenward by extending his arm, eventually losses its use by the unnatural and fixed rigidity of the joints, even as the fishes in the great Kentucky cave have, through long disuse, lost the faculty of sight, with only rudimentary specks in place of eyes. How many among us through a like disuse, have starved their vital forces into premature decay ?

All through nature, life means energy, activity and unceasing use, as surely as its opposite means disease and perpetual stagnation.

A man who follows the bent of a single purpose or idea, is the worst kind of a crank, and in failure, his state is pitiable for want of other resources mental and physical ; for he has ridden his own hobby to death ; his little light has gone out, and he becomes from thence a tax upon the patience of his fellows.

Not so with the man of culture. There is always room for him somewhere, and his adaptation to new conditions is as ready as it is secure, for he has taken pains to preserve a just equilibrium of all his faculties.

The expert athlete is careful not to cultivate any one part to the disparagement of another, since the weakness of a single member reduces the stronger ones to its level. Of what avail are strong arms with a weak spinal column, or robust legs with a thin, undeveloped chest ? All the vital organs must be strong, healthy and free of action, so dependent are they upon one another, and the same is measurably true of the mental faculties. A monomaniac is as useless and unreliable as a lunatic. The difference is only in degree, and neither is to be depended upon in an emergency, and the man with a single idea is of the same piece. The cure, or at least the preventive, is in the equal cultivation of the intellectual faculties, together with due and sufficient physical exercise, evenly distributed throughout the entire system. This, with pure air, nutritious food, regular and temperate habits, will ensure a life full of years and usefulness.

